# About how to capture and exploit the CO_2_ surplus that nature, per se, is not capable of fixing

**DOI:** 10.1111/1751-7915.12805

**Published:** 2017-08-14

**Authors:** Manuel S. Godoy, Beatrice Mongili, Debora Fino, M. Auxiliadora Prieto

**Affiliations:** ^1^ Polymer Biotechnology Lab Centro de Investigaciones Biologicas (CIB) C/Ramiro de Maeztu, 9 28040 Madrid Spain; ^2^ Department of Applied Science and Technology (DISAT) Politecnico di Torino Corso Duca Degli Abruzzi 24 Torino Italy

## Abstract

Human activity has been altering many ecological cycles for decades, disturbing the natural mechanisms which are responsible for re‐establishing the normal environmental balances. Probably, the most disrupted of these cycles is the cycle of carbon. In this context, many technologies have been developed for an efficient CO_2_ removal from the atmosphere. Once captured, it could be stored in large geological formations and other reservoirs like oceans. This strategy could present some environmental and economic problems. Alternately, CO_2_ can be transformed into carbonates or different added‐value products, such as biofuels and bioplastics, recycling CO_2_ from fossil fuel. Currently different methods are being studied in this field. We classified them into biological, inorganic and hybrid systems for CO_2_ transformation. To be environmentally compatible, they should be powered by renewable energy sources. Although hybrid systems are still incipient technologies, they have made great advances in the recent years. In this scenario, biotechnology is the spearhead of ambitious strategies to capture CO_2_ and reduce global warming.

The tremendous impacts of global warming are being felt all over the world due to humans’ unsustainable way of life. We have released to the atmosphere more CO_2_ than what nature has been capable of fixing. The best strategy requires very deep cuts in emissions, as well as the use of alternatives to fossil fuels around the world. In 2015, the United Nations Conference on Climate Change (COP21), for the first time in more than 20 years of United Nations negotiations, achieved a legally binding climate agreement, with the objective of maintaining global warming below 2°C. Meanwhile, different approaches are being implemented to diminish this environmental problem. In this study, we aimed to update the most promising biotechnological approaches to capture and exploit excess of CO_2_ to accomplish human beings quality of life integrated to the sustainability of our planet.

## CO_2_ capture and storage (CCS): sweeping the dust under the carpet?

**Figure 1:**
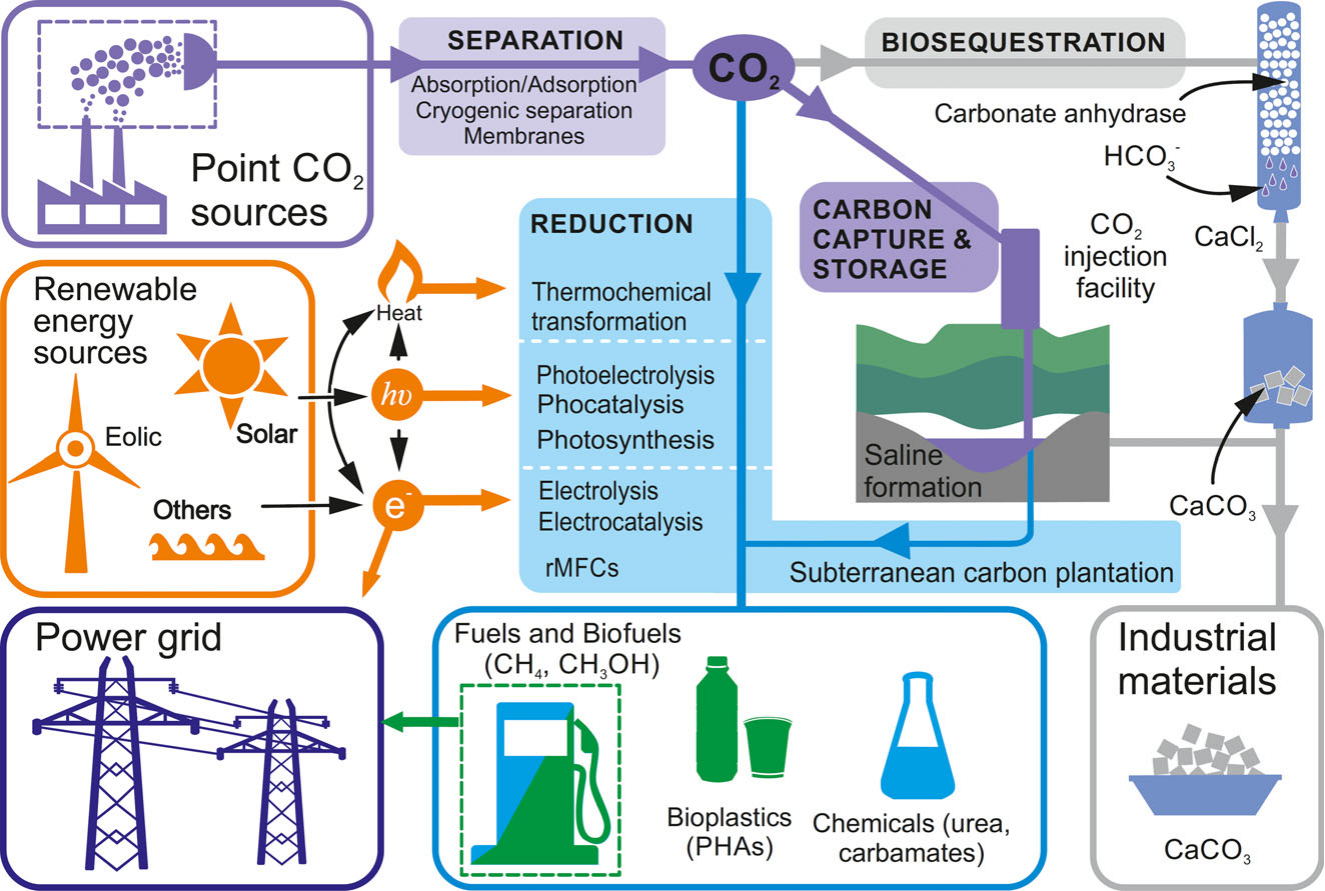
CO_2_ generated in point sources is separated by any of the existing methods (absorption/adsorption, cryogenic or membrane separation). The resulting almost pure CO_2_ can be captured and stored (carbon capture and storage – CCS) in geological formations (such as oil and gas fields, and saline formations) or transformed into different products. Carbonate can be produced using carbonate anhydrases from cyanobacteria and CaCl_2_ (biosequestration), and the resulting CaCO_3_ can be stored or used as precursors for construction materials. CO_2_ can also be reduced by different systems (biological, inorganic or hybrid). The energy supplied for the system to be sustainable must come from a renewable power source (wind, sun, etc.). This energy can be provided to the system as electrons (e‐), photons (*hv*) or heat. Subterranean carbon plantation is considered an alternative for recycling the CO_2_ captured in CCS into methane. The products of CO_2_ reduction can be bioplastics (PHAs), bio/fuels like methane or methanol. It can also be transformed into a wide range of chemicals (urea, carbamates, etc.). Fuels produced by CO_2_ reduction are a possible solution for the intermittence of renewable energy (power grid), as they can be stored using the existing facilities from fossil fuels.

One apparently promising technology is referred to as carbon capture and storage (CCS). CCS is a process consisting of the separation of CO_2_ from industrial and energy‐related sources, transport to a storage location and long‐term isolation from the atmosphere (Metz *et al*., 2005). Because the long‐distance transportation of CO_2_ to available disposal sites represents a prominent part of the economic and energetic costs of CO_2_ capture, it should be preferentially applied to large point sources. This includes large fossil fuel or biomass energy facilities, major CO_2_‐emitting industries, natural gas production, synthetic fuel plants and fossil fuel‐based hydrogen production plants (Aresta and Dibenedetto, 2007). Potential storage methods are geological storage (in geological formations, such as oil and gas fields, coal beds and deep saline formations), ocean storage (direct release into the ocean water column or onto the deep seafloor) and industrial fixation of CO_2_ into inorganic carbonates (Metz *et al*., 2005) (Fig. 1).

In this context, there are several commercially available technologies which can, in principle, be used for the separation of CO_2_ from the rest of the flue gases. However, the most employed method is a chemical absorption–desorption process, in which monoethanolamine solution is frequently used to dissolve the CO_2_. The almost pure CO_2_ is released afterwards from the liquid by heating to 100–150°C. The main drawback of this process is the requirement of intensive energy during regeneration of monoethanolamine solution. Other methods to separate CO_2_ include cryogenic fractionation, solid adsorption and membrane separation (Lam *et al*., 2012; Sreenivasulu *et al*., 2015).

The European Emissions Trading Scheme and the International Panel on Climate Change (IPCC) recognize that geological storage could be a valid mitigation option (West *et al*., 2005). In addition, the International Energy Agency (IEA) Blue Map scenario envisages a 19% CO_2_ reductions contribution from CCS by 2050 (Scott *et al*., 2012). However, there are some risks associated with this technology which must be considered. Although extensive physiological research is available, the environmental impacts of elevated CO_2_ on terrestrial, subsurface and marine ecosystems are not fully understood (West *et al*., 2005). Uncontrolled leakages could have implications for the environment. In economic terms, leaks into marine and freshwater systems might affect fisheries. For terrestrial systems, leakages might damage crops, groundwater quality and/or human and animal health. Other concerns include acidification, changes in biological diversity and species composition and asphyxiation at high CO_2_ concentrations. In addition, biogeochemical processes may be affected as increased CO_2_ concentrations could change pH, microbial populations and nutrient supply (Heinrich *et al*., 2003; Leung *et al*., 2014).

For this type of storage technology to be acceptable, safe practices must be developed, the potential for leaks must be understood, and dangerous situations must be avoided or safely managed (Lee *et al*., 2010). Other strategies, then, should be elaborated as alternative or, at least, to complement the geological and ocean storage of CO_2_. We will focus on the alternatives arising from microbiological world, especially prokaryotes, and to the contribution they can make to other inorganic technologies.

## Transforming greenhouse gases into added‐value products

As mentioned, final disposal of CO_2_ in different compartments (CCS) is a strategy to diminish its proportion in atmospheric gas composition. However, there are other strategies which are not just aiming at CO_2_ capture but also at its transformation and revalorization. A lot of research has been developed into this field. Mikkelsen and coworkers grouped CO_2_ transformations into different categories involving different chemical, physical or biological methods (Mikkelsen *et al*., 2010). To simplify, we will group the systems for CO_2_ transformation into three categories: (i) biological (which include living prokaryotes or enzymes); (ii) inorganic transformations (chemical, physical or a combination of both); and a third category (iii) hybrid systems in which the process of CO_2_ conversion covers biological and inorganic steps integrated into the same system.

## Biological systems for CO_2_ transformation

In the field of biological transformations, eukaryotic microalgae and cyanobacteria have been extensively studied for biofuels production and CO_2_ sequestration in carbonates via carbonic anhydrases (CAs). These organisms have been proposed for almost 50 years as a source of renewable fuels to reduce global warming (Oswald and Golueke, 1960).

Photosynthetic microorganisms grow 100 times faster than terrestrial plants, and they can double their biomass in <1 day. This is due to their simple cellular structure and large surface to volume ratio that give them the ability to uptake large amount of nutrients from water sources and thus promoting their growth rate. In addition, they can convert solar energy to chemical energy with efficiency of 10–50 times greater than terrestrial plants (Lam *et al*., 2012). A major appeal of photosynthetic microorganism cultures in greenhouse gas mitigation is that they must use concentrated forms of CO_2_, such as provided by power plant flue gases (Benemann, 2003).

Although the potential of microalgae and cyanobacteria to contribute to the world energy and commodities demand is high, there is a large gap between the current available technology and the one needed to supply the potential world demand. It is still necessary to solve a large number of bottlenecks related with biological, engineering and economic aspects (Acién *et al*., 2012). For example, photosynthetic microorganisms suffer inefficiencies arising from suboptimal light‐harvesting properties including the low energy capture and transfer efficiency of photosynthesis that will not be probably addressed in the near term (Khunjar *et al*., 2012; Torella *et al*., 2015). Possible solutions for this issue are discussed in the next sections.

### Exploiting CO_2_ concentrating mechanism of cyanobacteria

The decline in atmospheric CO_2_ levels and rising O_2_ were a selective pressure in the past that lead to compensate the inefficient Rubiscos from cyanobacteria, with complementary strategies for CO_2_ fixation. They had to develop an effective photosynthetic CO_2_ concentrating mechanism for improving carboxylation. This adaptation acts to raise the concentration of CO_2_ around Rubisco hence improving the efficiency of CO_2_‐fixation and providing a survival advantage in limiting CO_2_ environments. The main feature of the cyanobacterial CO_2_ concentrating mechanism is that cellular Rubisco is partitioned into a protein‐bound micro‐compartment, called the carboxysome, which allows an internal accumulation of CO_2_ with the contribution of carbonic anhydrase (Rae *et al*., 2011).

Carbonic anhydrase (CA) is an interesting option for CO_2_ sequestration coming from the biological world. These are found not only in cyanobacteria but in animals, plants and other microbes. CA catalyses a rapid transformation of CO_2_ and water to ${\text{HCO}}_{3}^{-}$ and protons. As CA has the highest catalytic efficiency for CO_2_ hydration (*k*cat ∼ 106 s^−1^), it is considered as prominent biocatalytic agent for CO_2_ sequestration technology developments. In presence of cations at modest pH *in vitro*, CA converts CO_2_ into CaCO_3_ (Kanth *et al*., 2013). CaCO_3_ is a common and thermodynamically stable mineral found in rocks worldwide and is the main component of shells of marine organisms, snails and eggs. If the widespread transformation of CO_2_ to CaCO_3_ is possible, it will represent a stable process for long‐term CO_2_ storage. The transformation of CO_2_ into carbonate compounds using biocatalysts in a biomimetic approach has advantages for thermodynamically stable CO_2_ storage as compared to other technologies. This approach does not need a monitoring system for potential leaks, as do CCS, and allows the reuse of carbonate compounds for building or industrial materials (Lee *et al*., 2010). There are still several problems for practical use of CA such as high cost, limited stability, narrow range of working pH, intoxication due to impurities in flue gas, various issues related to scaling up and operating conditions. So, more stable and high‐active CA should be screened or engineered for more practical CA‐catalysed CO_2_ sequestration system (Kanth *et al*., 2013).

Carboxysomes are not just interesting for microbiologists. Growth and productivity in important crop plants is limited by the inefficiencies of the C3 photosynthetic pathway. Introducing into C3 plants CO_2_‐concentrating mechanisms, such as carboxysomes, could overcome these limitations and lead to increased yields (Rae *et al*., 2017). Recently, different groups described the recombinant expression of synthetic carboxysome shells in *Escherichia coli* (Cai *et al*., 2016) and *Corynebacterium glutamicum* (Baumgart *et al*., 2017) as intermediate step before jumping onto plants for improved CO_2_ fixation and concomitant increased biomass production.

### Rational consortia between photoautotrophs and heterotrophs

As CO_2_ fixation tools, cyanobacteria can be co‐cultivated with other heterotrophic microorganisms providing them of reduced compounds from CO_2_. In this strategy, photosynthesis would provide organic carbon to an optimized heterotrophic organism (such as *E. coli*) which in turn would transform it into an added‐value compound. For example, the capability of sucrose‐secreting cyanobacteria to act as a platform for the construction of a light‐driven consortia was evaluated (Hays *et al*., 2017). In this work, the cyanobacteria *Synechococcus elongatus* PCC 7942 was paired with three disparate heterotrophs: *Bacillus subtilis*,* E. coli* or *Saccharomyces cerevisiae*. These synthetic consortia could be stabilized over the long term (weeks to months) and persisted in the face of selected perturbations (dilution, periods of darkness and phase changes in growth media). It could also be programmed for photoproduction of target compounds and proteins, such as alpha‐amylase and the bioplastic polyhydroxybutyrate (produced by co‐culturing cyanobacteria with recombinant *B. subtilis* or *E. coli* respectively). The principal advantage of this system is its modularity, and the wide range of products that could be synthesized by means of genetically flexible organisms associated with cyanobacteria.

### Natural and engineered CO_2_ fixation pathways

Today, six autotrophic CO_2_ fixation mechanisms are known: (i) the Calvin–Benson reductive pentose phosphate cycle; (ii) the reductive citric acid cycle (Arnon–Buchanan cycle); (iii) the reductive acetyl‐coA (Wood–Ljungdahl) pathway; (iv) the hydroxypropionate (Fuchs–Holo) bicycle (3‐hydroxypropionate cycle); (v) the 3‐hydroxypropionate/4‐hydroxybutyrate and (vi) the dicarboxylate/4‐hydroxybutyrate cycles (Berg, 2011). Many heterotrophs have been engineered so far with recombinant CO_2_ fixation enzymes and pathways (Parikh *et al*., 2006; Mueller‐Cajar and Whitney, 2008; Guadalupe‐Medina *et al*., 2013; Antonovsky *et al*., 2016). However, they still grow as mixotrophs, requiring an organic carbon source for the production of the starting substrates of linear CO_2_ fixation pathways and/or for the regeneration of ATP and/or electron donors. Converting these engineered heterotrophs into true autotrophs would require the functional transplantation of complete CO_2_ fixation cycles and the transplantation of, and integration with, energy‐harvesting systems (Claassens *et al*., 2016; Claassens, 2017).

To overcome limitations of the natural carbon fixation pathways, an extensive *in silico* study identified alternative pathways that combine existing metabolic building blocks from various organisms. This work suggested that some of the proposed synthetic pathways could have significant quantitative advantages over their natural counterparts, such as the overall kinetic rate (Bar‐Even *et al*., 2010). In an another attempt to improve CO_2_ fixation, Schwander *et al*. published the construction of a non‐natural optimized CO_2_ fixation cycle called the ‘crotonyl–coenzyme A (CoA)/ethylmalonyl‐CoA/hydroxybutyryl‐CoA’ (CETCH) cycle (Schwander *et al*., 2016). The 12 enzymes involved in the CETCH cycle come from six organisms across all three domains of life: one from a plant (*Arabidopsis thaliana*), one from humans (*Homo sapiens*) and the other 10 enzymes from microbes. Although the CETCH cycle has the fewest reactions and the lowest requirement for ATP and NADPH among the aerobic CO_2_ fixation pathways, it produces glyoxylate, a less reduced metabolic intermediate. As CETCH cycle functionality has only been demonstrated *in vitro*, it should be transplanted into a selected chassis organism to develop a whole‐cell biocatalyst (Gong and Li, 2016; Schwander *et al*., 2016).

## Inorganic transformations

The strategies for CO_2_ transformations can be grouped on the basis of the type of energy supplied to the CO_2 _for coping with its thermodynamic and kinetic stability (ΔG°f 396 kJ mol^−1^). They can be classified as (i) chemical and thermochemical or as (ii) photochemical and electrochemical. They will be summarized briefly here, but for more detailed reviews see references (Aresta and Dibenedetto, 2007; Mikkelsen *et al*., 2010).

### Chemical and thermochemical transformations

The chemical transformations include all those strategies getting the leverage over the little reactivity window offered by the CO_2_ structure. This transformation method is based on the electrophilicity of the central carbon and the electron‐rich behaviour of the two oxygen atoms hosted in the molecular structure. For this reason, the CO_2_ chemical reactions are usually associated with, for example, low‐valent metal complexes (e.g. Ni, Pa) (Sakakura *et al*., 2007). In addition, when the catalytic reactions are not able to promote the CO_2 _activation, the reactions are usually coupled with high temperature and pressure conditions (thermochemical conversion) and/or with high‐energy compounds like hydrogen (Mikkelsen *et al*., 2010).

The range of products reached by chemical conversion technologies is wide, encompassing both chemical carbon‐based compounds with high oxidation state (e.g. carbamates, urea and polymeric materials), and energy‐rich, and consequently, more reduced molecules, such as formic acid, formaldehyde, methanol, methane and other hydrocarbons (Mikkelsen *et al*., 2010; Liu *et al*., 2015b). However, despite this vast product scenario, currently the application of CO_2_ as a feedstock for industrial production is limited to the production of urea, cyclic carbonates, polymers and carbamates (Mikkelsen *et al*., 2010). When a catalyst or a chemical activator is not available, the CO_2_ can be converted under extremely high temperature and pressure conditions (Lou *et al*., 2003; Treacy and Ross, 2004).

### Photochemical and electrochemical

Leaf‐mimic system and artificial photosynthesis are alternative strategies for converting the CO_2_. These carbon fixation systems offer a way to avoid the high‐energy cost characterizing thermochemical conversions. They try to mimic the structural design of carbon catalyst centres or to reproduce the equivalent electron paths discovered in natural CO_2_ conversion systems (Berardi *et al*., 2014; Banerjee *et al*., 2016). Photochemical conversion methods are based on the exploitation of the sun‐light as energy source, a feature that renders this strategy sustainable for a further applications (Blankenship *et al*., 2011; Herron *et al*., 2015).

The artificial photosynthesis works towards a direct CO_2_ conversion for the synthesis of hydrocarbons or oxygenated products (Chang *et al*., 2016). The devices adopted for these methodologies are composed of the minimum components of natural photosynthetic systems, such as a light‐capturing antenna, a catalyst and an electron sacrificial donor compound. In this kind of devices, light promotes the electron transfer from the antenna system to the CO_2_, the catalyst favours a photo‐assisted multi‐electron transfer, and eventually, a sacrificial electron donor closes the electron cycle. The CO_2_ fixation devices are classified as photocatalytic system, if both reduction and oxidation occur in a same location, or as photo‐electrochemical cells if the redox reactions are spatially separated (Chang *et al*., 2016). In the latter, the redox reactions are carried out on the surface of two electrodes, which can be physically separated by a membrane (generally nafion) (Yim *et al*., 2015). The main products are generally represented by CO, formate and oxalate, even if also the synthesis of hydrocarbons, as methane or ethane, is attested (Jhong *et al*., 2013).

For electrochemical CO_2_ conversion, transition metals are the most used catalysts (Jhong *et al*., 2013). Among them, copper has the best selectivity to produce hydrocarbons and formic acid. It also offers the potentiality to catalyse the synthesis of C1‐, C2‐, C3‐based compounds and hydrocarbons with a faradic efficiency higher than 50% (Peterson and Nørskov, 2012). However, materials used for catalysing the reactions are not able to promote high values of reaction rate together with high selectivity, energetic efficiency and current density so far (Kuhl *et al*., 2012)(Montoya *et al*., 2017). Therefore, new cathode materials or modification of the catalyst surface is under investigation (Peterson and Nørskov, 2012).

## Hybrid systems: Integrating different technologies for the design of more efficient CO_2_ transforming systems

Hybrid systems for conversion of CO_2_ could be the solution for two different problems emerging from the battle against climate change; on one side, photosynthetic organisms suffer inefficiencies arising from non‐optimal light‐harvesting properties (Khunjar *et al*., 2012; Torella *et al*., 2015). In a typical fermentation process of sugar cane to ethanol, the final product contains only almost 0.2% of the available solar energy. This low efficiency is mainly due to ineffective plant photosynthesis, but also to energy losses in the subsequent processing of biomass and microbial fermentation. In comparison with biological photosynthesis, the efficiency of photovoltaic solar panels is very high; solar panels that are currently available have solar to electricity efficiencies around 18%, and new innovations may enable efficiencies of more than 40% (Claassens *et al*., 2016). By the other side, renewable energy sources have to deal with the problem of intermittence generation of electric‐power. In particular, the amount of solar radiation incident on the earth's surface depends upon many factors such as location (latitude), time of the day, inclination of the surface, declination and weather. Storing solar energy is critical for continuous processing during these fluctuations (Herron *et al*., 2015). As solar and wind penetration increases in our systems, the intermittency of these two energy sources seriously compromises the stability and quality of grid power (Tuller, 2017).

One option of energy storing could come for hydrogen technology. Water splitting powered by renewable energy sources can lead to the production of hydrogen as a fuel. Hydrogen has many attractive attributes – it is clean burning and can be efficiently converted back to electricity via fuel cells. Hydrogen lacks volumetric energy density, this is the amount of energy stored per unit volume, and cannot be easily stored and distributed like hydrocarbon fuels. Its utility is much greater as an onsite fuel for converting CO_2_ to CH_4_ or for generating heat, electricity or syngas (Tuller, 2017). Liquid fuels are more appealing as a solar storage medium because of their attractive energy density and existing sophisticated distribution and storage infrastructures. However, attempts to produce liquid fuel via inorganic CO_2_ reduction have generally poor specificity and energy efficiency (Torella *et al*., 2015).

**Figure 2:**
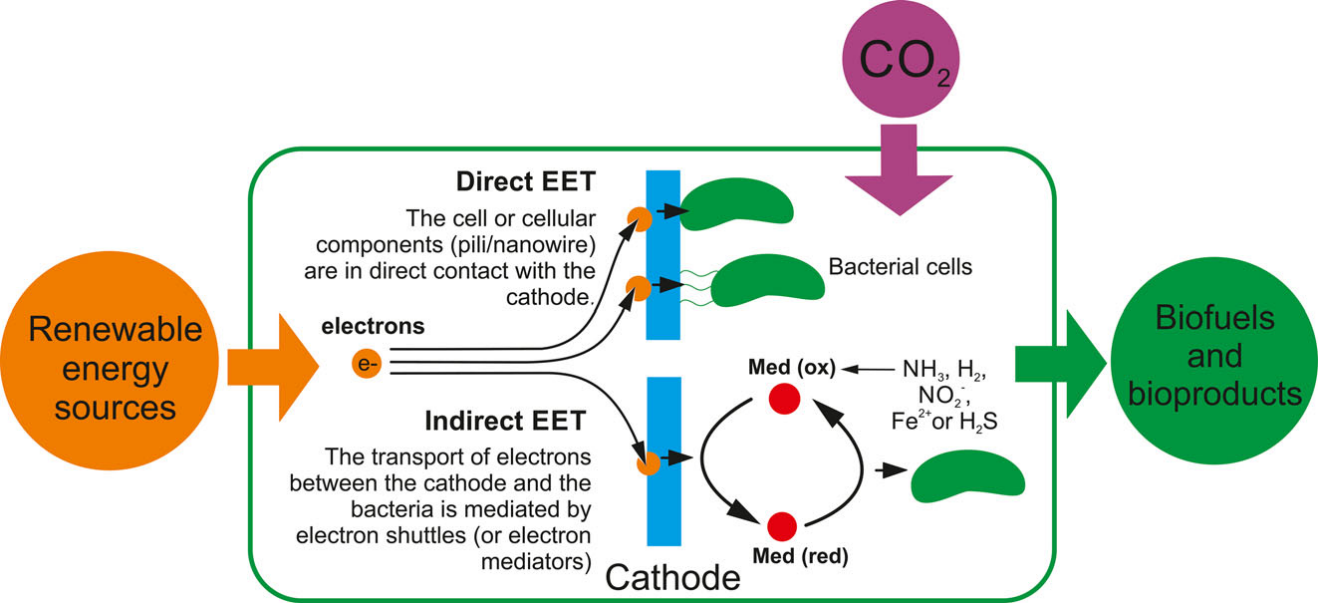
Schematic representation of CO_2_ reduction and extracellular electron transfer (EET) in hybrid systems. They are based on reverse microbial fuel cells and take advantage of the high CO_2_ reduction turnover of electrolysis and the versatility of microbial metabolism.

Thus, the problem of energy intermittence and low light‐harvesting properties of photosynthesis could be solved by integrating into a hybrid configuration the energy coming from renewable sources with biological systems as producers of storable fuels such as methane, alcohols, alkanes and other chemicals. This innovative technology is carried out in reverse microbial fuel cells and takes advantage of a higher CO_2_ reduction turnover from the electrochemical side and more interesting carbon products from the bio‐side. In a standard microbial fuel cell, organisms oxidize organic fuels and transfer electrons into an electrochemical system so that fuels are converted to electrical energy. In a reverse microbial fuel cells, this process is reversed so that electrical energy is used by bacterial cells to drive CO_2_ fixation to high‐energy organic compounds (Khunjar *et al*., 2012). The origin of these electrons may be any renewable energy source (sun, wind, etc.). The process of delivering these electrons to the cell is called extracellular electron transfer (EET). This transfer can be done in an indirect or direct fashion, depending whether they require or not the diffusion of a mobile component for electron transport (Rabaey and Rozendal, 2010) (Fig. 2).

### Indirect EET

In a reverse microbial fuel cells, the indirect method for EET involves the production or use of so‐called electron shuttles (or electron mediators), which transport the electrons from the electrode to the cell. The use of a mediator enables the utilization of planktonic cells in the bioreactor and can also enable separate‐stage designs that afford spatial and temporal decoupling of energy capture and bioproduction. This allows both processes to be operated and optimized separately. In the mediated approach, electrons are first transferred from the electrode to a soluble mediator, and then, the mediator is oxidized by the cell. Inorganic compounds that are linked with chemoautotrophy and can be electrolytically regenerated (such as NH_3_, H_2_, ${\text{NO}}_{2}^{-}$, Fe^2+^ and H_2_S), are an attractive options for this platform as they can facilitate the construction of multicarbon organics from CO_2_ using natural carbon fixation pathways (Khunjar *et al*., 2012).

Ammonia can be used as an indirect EET taking advantage of its low cost, abundance, safety and solubility. For example, *Nitrosomonas europaea*, a chemolithoautotroph, was used as the biocatalyst due to its inherent capability to utilize ammonia as its sole energy source for growth. Calculations indicated that overall production efficiency could approach approximately 2.7% under optimal electrolysis conditions. Considering a conversion efficiency of 10% from solar energy, the biomass production efficiency of this system was 0.27 ± 0.02%. This level of efficiency is comparable to photon to biomass conversion efficiencies observed for photosynthetic systems (around 1%) (Khunjar *et al*., 2012), so further efforts are required to improve these results, by considering more efficient conversions from solar energy, or modifying the genetic backgrounds of the strain.

*Ralstonia eutropha*, as a model chemolitoautotrophus species, is a promising tool for the development of hybrid systems. This bacterium is able to grow using electrochemically generated H_2_ as energy source. Engineered strain of *R. eutropha* H16 was studied for the production of isobutanol and 3‐methyl‐1‐butanol as the target fuels. Growth of *R. eutropha* was inhibited by reactive oxygen and nitrogen species when electric current was introduced. To circumvent this problem, a porous ceramic cup was used to shield the anode. This shield provided a tortuous diffusion path for chemicals. Therefore, the reactive compounds produced by the anode could be quenched before reaching the cells growing outside the cup. Using this approach, healthy growth of Ralstonia strain LH74D and production of over 140 mg l^−1^ biofuels were achieved with the electricity and CO_2_ as the sole source of energy and carbon respectively (Li *et al*., 2012). Based on electrochemically generated H_2_ as energy source*, R. eutropha* was also engineered for the production of fatty acid‐derived, diesel‐range methyl ketones. These modifications included overexpression of a cytoplasmic version of the TesA thioesterase, the deletion of two putative β‐oxidation operons and heterologous expression of three genes (the acyl coenzyme A oxidase gene from *Micrococcus luteus* and *fadB* and *fadM* from *E. coli*). These genetic modifications led to the production of 180 mg l^−1^ under chemolithoautotrophic growth conditions (Muller *et al*., 2013).

There are other ongoing projects using hybrid systems for CO_2_ capture. These projects aim to bridge a cost‐effective CO_2_ capture and purification, with electrochemical conversion of CO_2_, followed by the fermentation of the CO_2_‐reduction intermediates (such syngas and C1 water‐soluble molecules). By selecting the appropriate microorganism, a wide range of valuable products can be synthesized, such as PHAs, isoprene, lactic acid and methane (www.celbicon.org). For example, *Rhodospirillum rubrum*, a purple non‐sulfur bacterium, can produce PHAs from CO as carbon and energy source (Revelles *et al*., 2016). This feature and its metabolical versatility make this species interesting as biological tool for CO_2_ fixation.

### Direct EET

Direct transfer typically involves at least a series of periplasmic and outer membrane complexes. In recent years, the involvement of pili or pilus‐like appendages (called nanowires in this context) was established. It has been suggested that nanowires also establish electron transport between different microorganisms in a community (Rabaey and Rozendal, 2010). Nevin proposed the term ‘microbial electrosynthesis’ for the reduction of CO_2_ to multicarbon compounds with electrons donated from an electrode as the electron donor (Nevin *et al*., 2010).

The bioelectrochemical reduction of CO_2_ has been also postulated as a promising process to obtain methane. The term electromethanogenesis has been applied to the process of producing methane using CO_2_ as the sole carbon source, using electroactive microbes in an engineered system (biocathode) powered with electric current. CO_2_ can be fixed either by direct EET or indirect EET; however, we will mention examples of the former here. Electromethanogenesis‐based technologies have a great potential for storing renewable energy in the form of methane, improving waste treatment processes or upgrading gas streams containing CO_2_. In all cases, future studies must focus on further up‐scaling, increase process efficiencies and reduce operation costs to reach coexistence with well‐established technologies, or even a hypothetical overtaking (Blasco‐Gómez *et al*., 2017).

Electromethanogenesis can also add value to CO_2_ stored in geological formations by means of subterranean carbon plantation. This concept proposed by Sato and coworkers, is based on CCS technologies, *in situ* biological conversion of the stored CO_2_ to methane and harvest of the biogenic methane as a recycled energy source (Sato *et al*., 2013). When supplied with CO_2_ through CCS operations, such reservoirs could function as natural bioreactors that prompt methanogens to convert CO_2_ to methane. Intermittent electrical energy provided by, for instance, wind turbines and photovoltaic cells can be stored in a stable form as methane. The current limitation of the system is the relatively slow rate of electromethanogenesis, and further studies are required on electrochemical reduction of CO_2_ under more realistic conditions: at higher (hydrostatic) pressures, in presence of solid (rock) surfaces and in microbial symbioses (Kuramochi *et al*., 2013; Sato *et al*., 2013).

Methane may not be the only product of electrosynthesis. Using consortia of cathodophilic microorganisms from brewery wastewater, it is possible to obtain a mixture of products (acetate, methane and hydrogen) through electrosynthesis in the same process, with CO_2_ as the only carbon source. The electrochemical evidence suggests that the electron transfer between the electrode and microbes in a biofilm operates in the absence of soluble redox active components in the medium (Marshall *et al*., 2012). Liu and coworkers created a two‐step strategy that mimics natural photosynthesis, where light capture by a biocompatible nanowire array interfaced and directly provided reducing equivalents to living organisms. The high‐surface‐area silicon nanowire array harvests light energy to provide reducing equivalents to the anaerobic bacterium, *Sporomusa ovata*, for the photo‐electrochemical production of acetic acid under aerobic conditions (21% O_2_). The resulting acetate (∼6 g l^−1^) fed genetically engineered *E. coli* to produce a variety of value‐added chemicals. The yield of target molecules was as high as 26% for n‐butanol, 25% for one of the isoprenoid compounds (amorphadiene) and up to 52% for PHAs, comparable with literature values (Liu *et al*., 2015a).

## Concluding remarks

Global warming is an ongoing threat for the maintenance of ecological systems, for economy and human life quality. Solving the problem implies the convergence of different approaches to achieve a net CO_2_ removal from the atmosphere. CCS may account for part of the solution, but they must be complemented with other green technologies with lower environmental risks and higher economical sustainability. Microbial biotechnology can lend a great hand to this aim by providing recombinant microorganisms able to transform the low reactive molecule of CO_2_ into a wide variety of compounds, such as biofuels, bioplastics and chemicals. New hybrid technologies should also be explored to make the whole process more efficient. Electrochemical cells, as inorganic systems for CO2 fixation, have the potentiality to sequestrate high amounts of CO2, but their low selectivity and capacity for generating added‐value products are limitations in a commercial scenario. In contrast, biological systems are much more versatile for the production of C‐based molecules than electrochemical cells, but they are quite inefficient in harnessing solar energy. A promising strategy for sequestrate and fix CO_2_ is definitively based on the combination of bio and electrochemical systems.

## Conflict of interest

None declared.

## References

[mbt212805-bib-0001] Acién, F.G. , Fernández, J.M. , Magán, J.J. , and Molina, E. (2012) Production cost of a real microalgae production plant and strategies to reduce it. Biotechnol Adv 30: 1344–1353. 22361647 10.1016/j.biotechadv.2012.02.005

[mbt212805-bib-0002] Antonovsky, N. , Gleizer, S. , Noor, E. , Zohar, Y. , Herz, E. , Barenholz, U. , *et al* (2016) Sugar synthesis from CO_2_ in *Escherichia coli* . Cell 166: 115–125. 27345370 10.1016/j.cell.2016.05.064 PMC4930481

[mbt212805-bib-0003] Aresta, M. , and Dibenedetto, A. (2007) Utilisation of CO_2_ as a chemical feedstock: opportunities and challenges. Dalton Trans 0: 2975–2992. 10.1039/b700658f 17622414

[mbt212805-bib-0005] Banerjee, A. , Dick, G.R. , Yoshino, T. , and Kanan, M.W. (2016) Carbon dioxide utilization via carbonate‐promoted C‐H carboxylation. Nature 531: 215–219. 26961655 10.1038/nature17185

[mbt212805-bib-0006] Bar‐Even, A. , Noor, E. , Lewis, N.E. , and Milo, R. (2010) Design and analysis of synthetic carbon fixation pathways. Proc Natl Acad Sci 107: 8889–8894. 20410460 10.1073/pnas.0907176107 PMC2889323

[mbt212805-bib-0007] Baumgart, M. , Huber, I. , Abdollahzadeh, I. , Gensch, T. , and Frunzke, J. (2017) Heterologous expression of the Halothiobacillus neapolitanus carboxysomzral gene cluster in Corynebacterium glutamicum. J Biotechnol [Epub ahead of print] doi: 10.1016/j.jbiotec.2017.03.01. 10.1016/j.jbiotec.2017.03.019 28359868

[mbt212805-bib-0008] Benemann, J.R. (2003) Biofixation of CO_2_ and greenhouse gas abatement with microalgae – Technology roadmap. Final Rep US Dep Energy Natl Energy Technol Lab 1–29.

[mbt212805-bib-0009] Berardi, S. , Drouet, S. , Francàs, L. , Gimbert‐Suriñach, C. , Guttentag, M. , Richmond, C. , *et al* (2014) Molecular artificial photosynthesis. Chem Soc Rev 43: 7501–7519. 24473472 10.1039/c3cs60405e

[mbt212805-bib-0010] Berg, I.A. (2011) Ecological aspects of the distribution of different autotrophic CO_2_ fixation pathways. Appl Environ Microbiol 77: 1925–1936. 21216907 10.1128/AEM.02473-10 PMC3067309

[mbt212805-bib-0011] Blankenship, R.E. , Tiede, D.M. , Barber, J. , Brudvig, G.W. , Fleming, G. , Ghirardi, M. , *et al* (2011) Comparing photosynthetic and photovoltaic efficiencies and recognizing the potential for improvement. Science 332: 805–809. 21566184 10.1126/science.1200165

[mbt212805-bib-0012] Blasco‐Gómez, R. , Batlle‐Vilanova, P. , Villano, M. , Balaguer, M. , Colprim, J. , and Puig, S. (2017) On the edge of research and technological application: a critical review of electromethanogenesis. Int J Mol Sci 18: 874. 10.3390/ijms18040874 PMC5412455 28425974

[mbt212805-bib-0013] Cai, F. , Bernstein, S.L. , Wilson, S.C. , and Kerfeld, C.A. (2016) Production and characterization of synthetic carboxysome shells with incorporated luminal proteins. Plant Physiol 170: 1868–1877. 26792123 10.1104/pp.15.01822 PMC4775138

[mbt212805-bib-0014] Chang, X. , Wang, T. , and Gong, J. (2016) CO_2_ photo‐reduction: insights into CO_2_ activation and reaction on surfaces of photocatalysts. Energy Environ Sci 9: 2177–2196.

[mbt212805-bib-0015] Claassens, N.J. , Sousa, D.Z. , dos Santos, V.A.P.M. , de Vos, W.M. , and van der Oost, J. (2016) Harnessing the power of microbial autotrophy. Nat Rev Microbiol 14: 692–706. 27665719 10.1038/nrmicro.2016.130

[mbt212805-bib-0501] Claassens, N. J. (2017). A warm welcome for alternative CO2 fixation pathways in microbial biotechnology. Microb. Biotechnol., 10, 31–34. 27873465 10.1111/1751-7915.12456 PMC5270723

[mbt212805-bib-0016] Gong, F. , and Li, Y. (2016) Fixing carbon, unnaturally. Science 354: 830–831. 27856865 10.1126/science.aal1559

[mbt212805-bib-0017] Guadalupe‐Medina, V. , Wisselink, H. , Luttik, M.A. , de Hulster, E. , Daran, J.‐M. , Pronk, J.T. , and van Maris, A.J. (2013) Carbon dioxide fixation by Calvin‐Cycle enzymes improves ethanol yield in yeast. Biotechnol Biofuels 6: 125. 23987569 10.1186/1754-6834-6-125 PMC3766054

[mbt212805-bib-0018] Hays, S.G. , Yan, L.L.W. , Silver, P.A. , and Ducat, D.C. (2017) Synthetic photosynthetic consortia define interactions leading to robustness and photoproduction. J Biol Eng 11: 4. 28127397 10.1186/s13036-017-0048-5 PMC5259876

[mbt212805-bib-0019] Heinrich, J.J. , Herzog, H.J. and Reiner, D.M. (2003) Environmental assessment of geologic storage of CO_2_ . In *Second National Conference on Carbon Sequestration* Citeseer, pp. 5–8.

[mbt212805-bib-0020] Herron, J.A. , Kim, J. , Upadhye, A.A. , Huber, G.W. , and Maravelias, C.T. (2015) A general framework for the assessment of solar fuel technologies. Energy Environ Sci 8: 126–157.

[mbt212805-bib-0021] Jhong, H.M. , Ma, S. , and Kenis, P.J.A. (2013) Electrochemical conversion of CO_2_ to useful chemicals : current status, remaining challenges, and future opportunities. Curr Opin Chem Eng 2: 191–199.

[mbt212805-bib-0022] Kanth, B.K. , Lee, J. , and Pack, S.P. (2013) Carbonic anhydrase : Its biocatalytic mechanisms and functional properties for efficient CO_2_ capture process development. Eng Life Sci 1: 422–431.

[mbt212805-bib-0023] Khunjar, W.O. , Sahin, A. , West, A.C. , Chandran, K. , and Banta, S. (2012) Biomass production from electricity using ammonia as an electron carrier in a reverse microbial fuel cell. PLoS ONE 7: e44846. 23028643 10.1371/journal.pone.0044846 PMC3446996

[mbt212805-bib-0024] Kuhl, K.P. , Cave, E.R. , Abram, D.N. , and Jaramillo, T.F. (2012) New insights into the electrochemical reduction of carbon dioxide on metallic copper surfaces. Energy Environ Sci 5: 7050–7059.

[mbt212805-bib-0025] Kuramochi, Y. , Fu, Q. , Kobayashi, H. , Ikarashi, M. , Wakayama, T. , Kawaguchi, H. , *et al* (2013) Electromethanogenic CO_2_ conversion by subsurface‐reservoir microorganisms. Energy Procedia 37: 7014–7020.

[mbt212805-bib-0026] Lam, M.K. , Lee, K.T. , and Mohamed, A.R. (2012) Current status and challenges on microalgae‐based carbon capture. Int J Greenhouse Gas Control 10: 456–469.

[mbt212805-bib-0027] Lee, S.W. , Park, S.B. , Jeong, S.K. , Lim, K.S. , Lee, S.H. , and Trachtenberg, M.C. (2010) On carbon dioxide storage based on biomineralization strategies. Micron 41: 273–282. 20144548 10.1016/j.micron.2009.11.012

[mbt212805-bib-0028] Leung, D.Y.C. , Caramanna, G. , and Maroto‐Valer, M.M. (2014) An overview of current status of carbon dioxide capture and storage technologies. Renew Sustain Energy Rev 39: 426–443.

[mbt212805-bib-0029] Li, H. , Opgenorth, P.H. , Wernick, D.G. , Rogers, S. , Wu, T.‐Y. , Higashide, W. , *et al* (2012) Integrated electromicrobial conversion of CO_2_ to higher alcohols. Science 335: 1596. 22461604 10.1126/science.1217643

[mbt212805-bib-0030] Liu, C. , Gallagher, J.J. , Sakimoto, K.K. , Nichols, E.M. , Chang, C.J. , Chang, M.C.Y. , and Yang, P. (2015a) Nanowire‐bacteria hybrids for unassisted solar carbon dioxide fixation to value‐added chemicals. Nano Lett 15: 3634–3639. 25848808 10.1021/acs.nanolett.5b01254 PMC5812269

[mbt212805-bib-0031] Liu, Q. , Wu, L. , Jackstell, R. , and Beller, M. (2015b) Using carbon dioxide as a building block in organic synthesis. Nat Commun 6: 5933. 25600683 10.1038/ncomms6933

[mbt212805-bib-0032] Lou, Z. , Chen, Q. , Zhang, Y. , Wang, W. , and Qian, Y. (2003) Diamond Formation by Reduction of Carbon Dioxide at Low Temperatures. J. Am. Chem. Soc 125: 9302–9303. 12889953 10.1021/ja035177i

[mbt212805-bib-0033] Marshall, C.W. , Ross, D.E. , Fichot, E.B. , Norman, R.S. , and May, H.D. (2012) Electrosynthesis of commodity chemicals by an autotrophic microbial community. Appl Environ Microbiol 78: 8412–8420. 23001672 10.1128/AEM.02401-12 PMC3497389

[mbt212805-bib-0034] Metz, B. , Davidson, O. , de Coninck, H. , Loos, M. and Meyer, L.M. (eds.) (2005) IPCC special report on carbon dioxide capture and storage. New York, NY: Cambridge University Press.

[mbt212805-bib-0035] Mikkelsen, M. , Jørgensen, M. , and Krebs, F.C. (2010) The teraton challenge. A review of fixation and transformation of carbon dioxide. Energy Environ Sci 3: 43–81.

[mbt212805-bib-0036] Montoya, J.H. , Seitz, L.C. , Chakthranont, P. , Vojvodic, A. , Jaramillo, T.F. , and Nørskov, J.K. (2017) Materials for solar fuels and chemicals. Nat Mater 16: 70–81. 10.1038/nmat4778 27994241

[mbt212805-bib-0037] Mueller‐Cajar, O. , and Whitney, S. (2008) Evolving improved Synechococcus Rubisco functional expression in *Escherichia coli* . Biochem J 414: 205–214. 18484948 10.1042/BJ20080668

[mbt212805-bib-0038] Muller, J. , MacEachran, D. , Burd, H. , Sathitsuksanoh, N. , Bi, C. , Yeh, Y.‐C. , *et al* (2013) Engineering of Ralstonia eutropha H16 for autotrophic and heterotrophic production of methyl ketones. Appl Environ Microbiol 79: 4433–4439. 23686271 10.1128/AEM.00973-13 PMC3697500

[mbt212805-bib-0039] Nevin, K.P. , Woodard, T.L. , and Franks, A.E. (2010) Microbial electrosynthesis: feeding microbes electricity to convert carbon dioxide and water to multicarbon extracellular organic compounds. MBio 1: e00103–e00110. 20714445 10.1128/mBio.00103-10 PMC2921159

[mbt212805-bib-0040] Oswald, W.J. , and Golueke, C.G. (1960) Biological transformation of solar energy. Adv Appl Microbiol 2: 223–262. 13731568 10.1016/s0065-2164(08)70127-8

[mbt212805-bib-0041] Parikh, M.R. , Greene, D.N. , Woods, K.K. , and Matsumura, I. (2006) Directed evolution of RuBisCO hypermorphs through genetic selection in engineered E.coli. Protein Eng Des Sel 19: 113–119. 16423843 10.1093/protein/gzj010 PMC2012944

[mbt212805-bib-0042] Peterson, A. , and Nørskov, J. (2012) Activity descriptors for CO_2_ electroreduction to methane on transition metal catalysts. J Phys Chem Lett 3: 251–258.

[mbt212805-bib-0043] Rabaey, K. , and Rozendal, R.A. (2010) Microbial electrosynthesis – revisiting the electrical route for microbial production. Nat Rev Microbiol 8: 706–716. 20844557 10.1038/nrmicro2422

[mbt212805-bib-0044] Rae, B.D. , Förster, B. , Badger, M.R. , and Price, G.D. (2011) The CO_2_‐concentrating mechanism of Synechococcus WH5701 is composed of native and horizontally‐acquired components. Photosynth Res 109: 59–72. 21384181 10.1007/s11120-011-9641-5

[mbt212805-bib-0045] Rae, B.D. , Long, B.M. , Förster, B. , Nguyen, N.D. , Velanis, C.N. , Atkinson, N. , *et al* (2017) Progress and challenges of engineering a biophysical carbon dioxide‐concentrating mechanism into higher plants. J Exp Bot 1–21. 28444330 10.1093/jxb/erx133

[mbt212805-bib-0046] Revelles, O. , Tarazona, N. , García, J.L. , and Prieto, M.A. (2016) Carbon roadmap from syngas to polyhydroxyalkanoates in *Rhodospirillum rubrum* . Environ Microbiol 18: 708–720. 26472698 10.1111/1462-2920.13087

[mbt212805-bib-0047] Sakakura, T. , Choi, J. , and Yasuda, H. (2007) Transformation of carbon dioxide. Chem Rev 107: 2365–2387. 17564481 10.1021/cr068357u

[mbt212805-bib-0048] Sato, K. , Kawaguchi, H. , and Kobayashi, H. (2013) Bio‐electrochemical conversion of carbon dioxide to methane in geological storage reservoirs. Energy Convers Manag 66: 343–350.

[mbt212805-bib-0049] Schwander, T. , Schada von Borzyskowski, L. , Burgener, S. , Cortina, N.S. , and Erb, T.J. (2016) A synthetic pathway for the fixation of carbon dioxide *in vitro* . Science 354: 900–904. 27856910 10.1126/science.aah5237 PMC5892708

[mbt212805-bib-0050] Scott, V. , Gilfillan, S. , Markusson, N. , Chalmers, H. , and Haszeldine, R.S. (2012) Last chance for carbon capture and storage. Nat Clim Chang 3: 105–111.

[mbt212805-bib-0051] Sreenivasulu, B. , Gayatri, D.V. , Sreedhar, I. , and Raghavan, K.V. (2015) A journey into the process and engineering aspects of carbon capture technologies. Renew Sustain Energy Rev 41: 1324–1350.

[mbt212805-bib-0052] Torella, J.P. , Gagliardi, C.J. , Chen, J.S. , Bediako, D.K. , Colón, B. , Way, J.C. , *et al* (2015) Efficient solar‐to‐fuels production from a hybrid microbial–water‐splitting catalyst system. Proc Natl Acad Sci 112: 201503606. 10.1073/pnas.1424872112 PMC4345567 25675518

[mbt212805-bib-0053] Treacy, D. , and Ross, J.R.H. (2004) The potential of the CO_2_ reforming of CH_4_ as a method of CO_2_ mitigation. A thermodynamic study. Am Chem Soc Div Fuel Chem 49: 126–127.

[mbt212805-bib-0054] Tuller, H.L. (2017) Solar to fuels conversion technologies: a perspective. Mater Renew Sustain Energy 6: 3. 28203516 10.1007/s40243-017-0088-2 PMC5283507

[mbt212805-bib-0055] West, J.M. , Pearce, J. , Bentham, M. , and Maul, P. (2005) Environmental issues and the geological storage of CO_2_ . Eur Environ 15: 250–259.

[mbt212805-bib-0056] Yim, K.‐J. , Song, D.‐K. , Kim, C.‐S. , Kim, N.‐G. , Iwaki, T. , Ogi, T. , *et al* (2015) Selective, high efficiency reduction of CO_2_ in a non‐diaphragm‐based electrochemical system at low applied voltage. RSC Adv 5: 9278–9282.

